# The Application of Ultrasonic Vibration in Human Sperm Cryopreservation as a Novel Method for the Modification of Physicochemical Characteristics of Freezing Media

**DOI:** 10.1038/s41598-019-46424-0

**Published:** 2019-07-11

**Authors:** Gholami Dariush, Riazi Gholamhossein, Fathi Rouhollah, Ghaffari Seyed Mahmood, Shahverdi Abdolhossein, Sharafi Mohsen, Alaei Loghman

**Affiliations:** 1 Institute of Biochemistry and Biophysics (IBB), University of Tehran, Tehran, Iran; 2 Department of Embryology, Reproduction Biomedicine Research Center, Royan Institute for Reproductive Biomedicine, ACER, Tehran, Iran; 3 Department of Poultry Sciences, Faculty of Agriculture, Tarbiat Modares University, Tehran, Iran

**Keywords:** Biological techniques, Embryology, Molecular medicine

## Abstract

The application of ultrasonic vibration was performed to modify the water molecules as the main compositions of the freezing medium used for human sperm cryopreservation. Different time periods of ultrasonic vibration (ULV) at the frequency of 28 kHz were applied for the evaluation of physicochemical properties of the water molecules. The most significant bubble size, zeta potential, and pH were obtained for the water molecules exposed to ultrasonic vibrations for 18 minutes and this time period was selected for further experiments due to the optimum results. In the next stage, semen samples were diluted with freezing medium containing ULV-exposed water and then cryopreserved. All the semen parameters were significantly reduced in cryopreserved groups as compared with the fresh control group. The highest percentage of total and progressive motility, viability, membrane and DNA integrity, and mitochondrial membrane potential were observed in frozen ULV compared with the frozen control. The rate of apoptosis in frozen ULV was significantly lower than that of in the frozen control. Furthermore, the gene expression ratios of α- and β-tubulins were significantly increased during cryopreservation, while the expression ratio of the tubulin polymerization promoting protein (TPPP) gene was decreased. Similar results were also observed when the protein levels of the genes mentioned earlier were evaluated by the ELISA method. Therefore, the changes in physicochemical properties of the freezing medium of human sperm cryopreservation using ULV can improve the quality of frozen products.

## Introduction

Cryopreservation allows the long-term storage of sperm at very low temperatures, preventing cellular aging through metabolic arrest and it preserves the viability and fertility potential of sperm for an extended period^1^. This process leads to fertility preservation in male patients who undergo chemotherapy and radiotherapy, resulted in testicular dysfunction^2^. However, cryopreservation has detrimental effects on structural, biochemical, and functional properties of sperm, mediated by the intracellular and extracellular crystal ice formation which could affect the fertility potential of thawed sperm^3–6^. Considering the arrangement of the water molecules is closely correlated with the harmful impacts of cryopreservation, the formation of hexagonal ice crystals can influence the water molecules structure, leading to the induction of reactive oxygen species (ROS) production, severe damage to the sperm membrane, and loss of sperm survival. Therefore, disturbance in the regular network of the water molecules, as well as the prevention of the formation of undesirable crystals may hinder further damages to the cells^7^. Physicochemical characteristics of the water such as viscosity, surface tension, pH, electric conductivity, and zeta potential are thought to be affected by external factors including temperature, magnetic fields, electromagnetic fields, and ultrasound^8–12^.

Therefore, in the present study, we employed a new strategy to interfere with the regular network of the water molecules to halt the process of crystal production in the cryopreservation medium. Upon the exposure of the water molecules to ULV in different periods, the optimal water sample was chosen for further experiments. In the second experiment, semen samples were cryopreserved by the ULV-exposed freezing medium, and then motion characteristics, viability, membrane integrity, acrosome integrity, apoptotic status, membrane mitochondria potential, DNA fragmentation index (DFI), and ROS were assessed. Furthermore, the expression ratios of α, β, and γ tubulins, as well as the expression of TPPP at the levels of mRNA and protein, were carried out during cryopreservation.

The current study aimed to disrupt the regular network array of the water molecules utilizing ULV to induce cavitation formations in the water^12^ and changes in the shape of ice crystal during human semen cryopreservation.

## Results

### Measurement of the mean bubble size and electric conductivity

**Figure 1:**
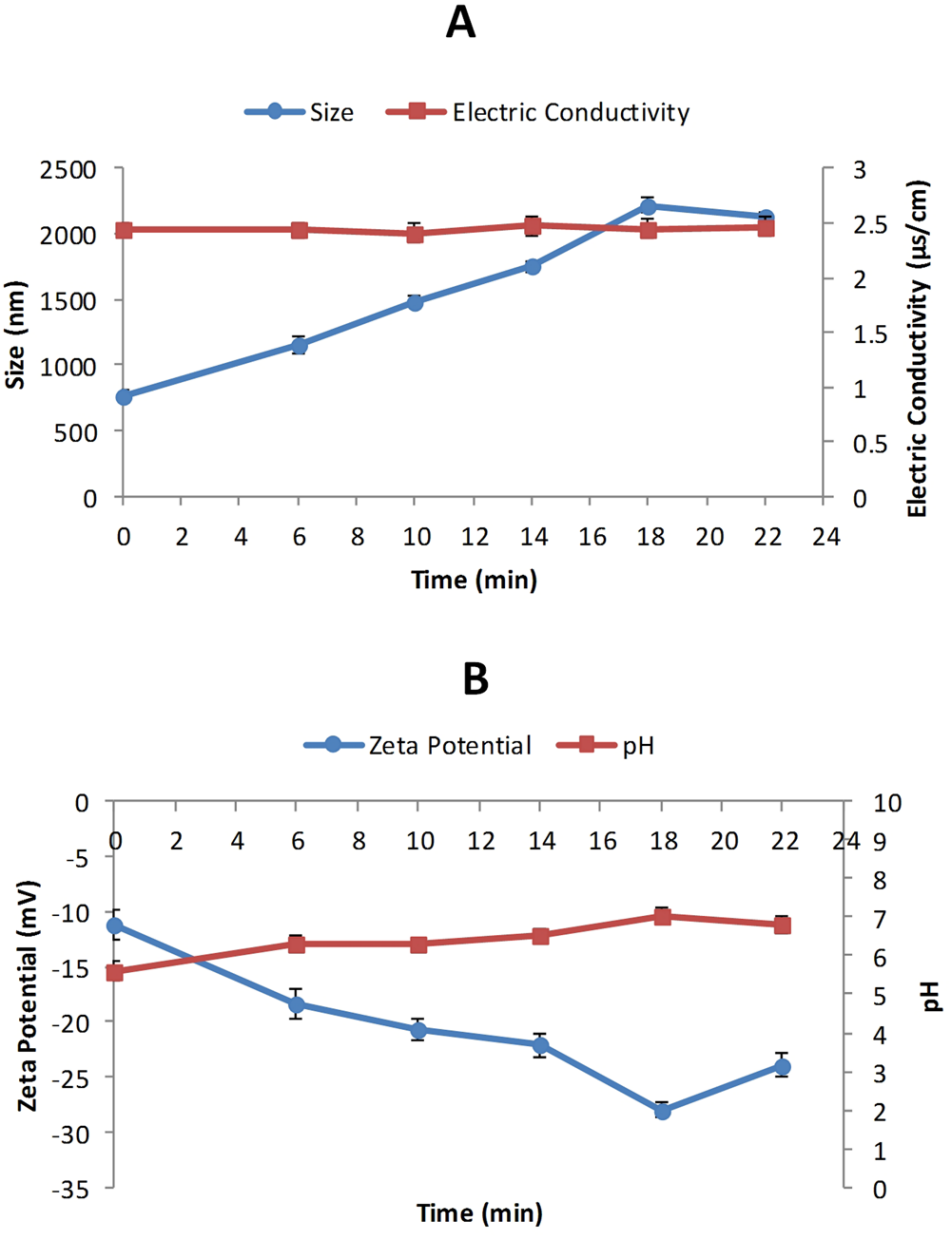
Physicochemical properties of the water molecules. (**A**) The bubble size was markedly influenced by the employment of ULV for the water molecules in different time periods. The electric conductivity remained unchanged in response to ULV exposure. (**B**) The average zeta potential values of ULV-exposed water molecules was considerably affected during various time periods. The highest pH value was observed in ULV-exposed water molecules with the time period of 18 minutes. Values are expressed as the mean and standard deviation (mean ± SD).

The mean bubble size was significantly (p < 0.001) decreased in parallel with an increase in the time periods. As shown in Fig. 1A, the highest mean bubble size (2218.70 ± 41.74 nm) was observed in ULV-exposed water molecules with the time period of 18 minutes as compared with other samples with the following time periods: 0 minute (767.95 ± 5.58 nm), 6 minutes (1157.68 ± 22.82 nm), 10 minutes (1486.94 ± 20.14 nm), 14 minutes (1751.81 ± 15.90 nm), and 22 minutes (2125.45 ± 42.79 nm). The lowest mean bubble size was found in a 6 minutes time period. Electric conductivity was not significantly (p > 0.001) different in all various time periods.

### Zeta potential and pH measurements

The impacts of variable time periods showed significant (p < 0.001) differences in zeta potential characteristics of sperm. As depicted in Fig. 1B, the highest average zeta potential (negative or positive values) was found in samples subjected to ULV for 18 minutes (−28.01 ± 0.75 mV) in comparison with the time periods of 0 minute (−11.20 ± 1.35 mV), 6 minutes (−18.40 ± 1.35 mV), 10 minutes (−20.72 ± 1.02 mV), 14 minutes (−22.16 ± 0.97 mV), and 22 minutes (−23.92 ± 1.15 mV).

As displayed in Fig. 1B, the pH of the water was increased after being exposed to ULV. The highest increase in the pH of the water was recorded in samples treated with ULV for 18 minutes (7.04 ± 0.19) in comparison to samples exposed to ULV for 0 minutes (5.6 ± 0.23), 6 minutes 6.32 ± 0.18), 10 minutes (6.29 ± 0.18), 14 minutes (6.54 ± 0.17), and 22 minutes (6.82 ± 0.23). The lowest rate of increment was detected in specimens subjected to ULV for 6 minutes.

### Motion characteristics

Post-thaw sperm parameters and motion variables in fresh control, frozen control, and frozen ULV are presented in Table 1. Total and progressive motility were significantly (p < 0.001) higher in the fresh control and frozen ULV than that of the frozen control group. In addition, total and progressive motility were significantly (p < 0.001) increased in the fresh control compared with the frozen ULV. Moreover, curvilinear velocity (VCL), straight linear velocity (VSL), and average path velocity (VAP) were significantly (p < 0.001) elevated in fresh control and frozen ULV in comparison with frozen control. These variables were significantly (p < 0.001) higher in the fresh control as compared with the frozen ULV. The linearity (LIN) motion variable was considerably higher in the fresh control than that of the frozen control and frozen ULV while this variable was not significantly different between the frozen control and frozen ULV (p > 0.001). The straightness (STR), amplitude of lateral head displacement (ALH), and beat-cross frequency (BCF) were dramatically increased in the fresh control group as compared with the frozen control. These variables were not significantly different in all groups (p > 0.001).

**Table 1:** Sperm quality parameters.

Characteristics	Groups		
	Fresh Control (n = 25)	Frozen Control (n = 25)	Frozen ULV (n = 25)
Total motility (%)	75.20 ± 2.06^a^	31.83 ± 0.93^c^	50.16 ± 0.97^b^
Progressive (%)	48.20 ± 3.3^a^	10.29 ± 0.65^c^	23.65 ± 1.2^b^
Non-progressive (%)	27.00 ± 2.21	21.54 ± 0.77	26.51 ± 1.21
**Motion variables**			
VCL (µm/s)	82.32 ± 6.68^a^	41.12 ± 2.60^c^	57.73 ± 3.38^b^
VSL (µm/s)	41.78 ± 4.01^a^	14.54 ± 1.33^c^	24.63 ± 2.1^b^
VAP (µm/s)	52.97 ± 4.44^a^	21.19 ± 1.58^c^	33.17 ± 2.42^b^
LIN (%)	49.19 ± 2.15^a^	34.74 ± 2.21^b^	40.94 ± 2.32^b^
STR (%)	76.09 ± 2.04^a^	66.40 ± 2.66^b^	71.08 ± 2.64^ab^
ALH (µM)	2.29 ± 0.14^a^	1.74 ± 0.13^b^	1.97 ± 0.1^ab^
BCF (Hz)	16.1 ± 0.95^a^	11.99 ± 1.31^b^	13.67 ± 1.08^ab^
**Other data**			
Extracellular ROS (RLU/Sec/10^6^sperm)	18.80 ± 0.54^c^	55.48 ± 1.28^a^	35.24 ± 0.38^b^
DCFH-DA Level (%)	7.42 ± 0.82^c^	33.27 ± 2.15^a^	16.25 ± 1.32^b^
DHE Level (%)	6.00 ± 0.59^c^	31.64 ± 2.12^a^	12.24 ± 1.21^b^
Seminal MDA (nmol/ml)	22.64 ± 0.47^b^	28.78 ± 0.49^a^	23.67 ± 0.33^b^

Total and progressive motility, sperm motion variables, seminal MDA, and ROS. The following assigned letters a, b and c denote significant differences (P < 0.001) among the experimental groups. Values are expressed as the mean and standard error of the mean (mean ± SEM); Tukey’s post hoc test.

### Viability and morphology

**Figure 2:**
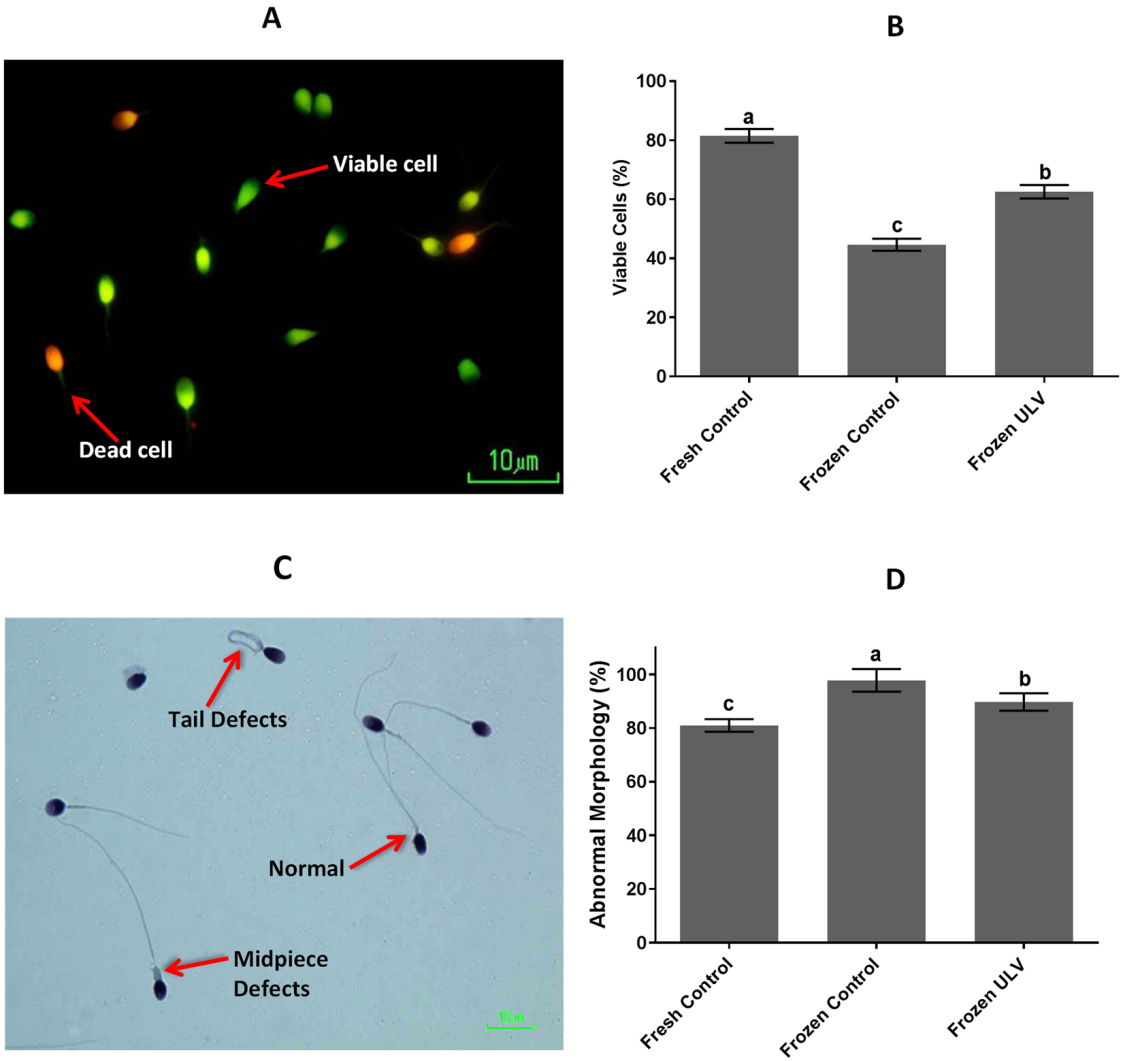
The viability and morphology of sperm. (**A**) The viable (green) and dead (red) sperm were stained with SYBR14/PI. (**B**) The percentage of viable cells was significantly altered when groups were compared with each other. (**C**) The morphology of sperm stained with Papanicolaou is evident. (**D**) The percentage of sperm with abnormal morphology was markedly influenced by the exposure to ULV among experimental groups. The following letters a, b and c indicate significant differences when the groups were compared with each other. Values are expressed as the mean ± SD; Tukey’s post hoc test; n = 25.

Figure 2 displays the percentage of the sperm viability and morphology of all experimental groups. The rate of viability was substantially higher (p < 0.001) in the frozen ULV compared with the frozen control (62.54 ± 2.29 vs. 44.59 ± 2.05%, respectively). This value was significantly higher in the fresh control (81.48 ± 2.32%; P < 0.001) as compared with the frozen control and frozen ULV. In addition, the number of sperm with abnormal morphology was significantly lower in the fresh control (80.96 ± 2.33%) in comparison with the frozen control (97.76 ± 4.18%) and frozen ULV (89.72 ± 3.25%). This value was considerably (p < 0.001) lower in the frozen ULV compared with the frozen control.

### Measurement of ROS, and MDA

As shown in Table 1, the extracellular ROS production in the frozen control was significantly (p < 0.001) increased in the fresh control and frozen ULV. This index was significantly (p < 0.001) higher in frozen ULV than that of the fresh control. Besides, a higher significant positive signal of DCFH was detected in the frozen control compared with the fresh and frozen ULV. The percentage of dihydroethidium (DHE) in the frozen control was significantly (p < 0.001) elevated in the frozen control compared with the fresh control and frozen ULV.

The malondialdehyde (MDA) levels of the seminal plasma were significantly (p < 0.001) decreased in the fresh control and frozen ULV as compared with the frozen control. This value did not significantly change (p < 0.001) between fresh control and frozen ULV (Table 1).

### Membrane integrity, acrosome integrity, and mitochondrial membrane potential

**Figure 3:**
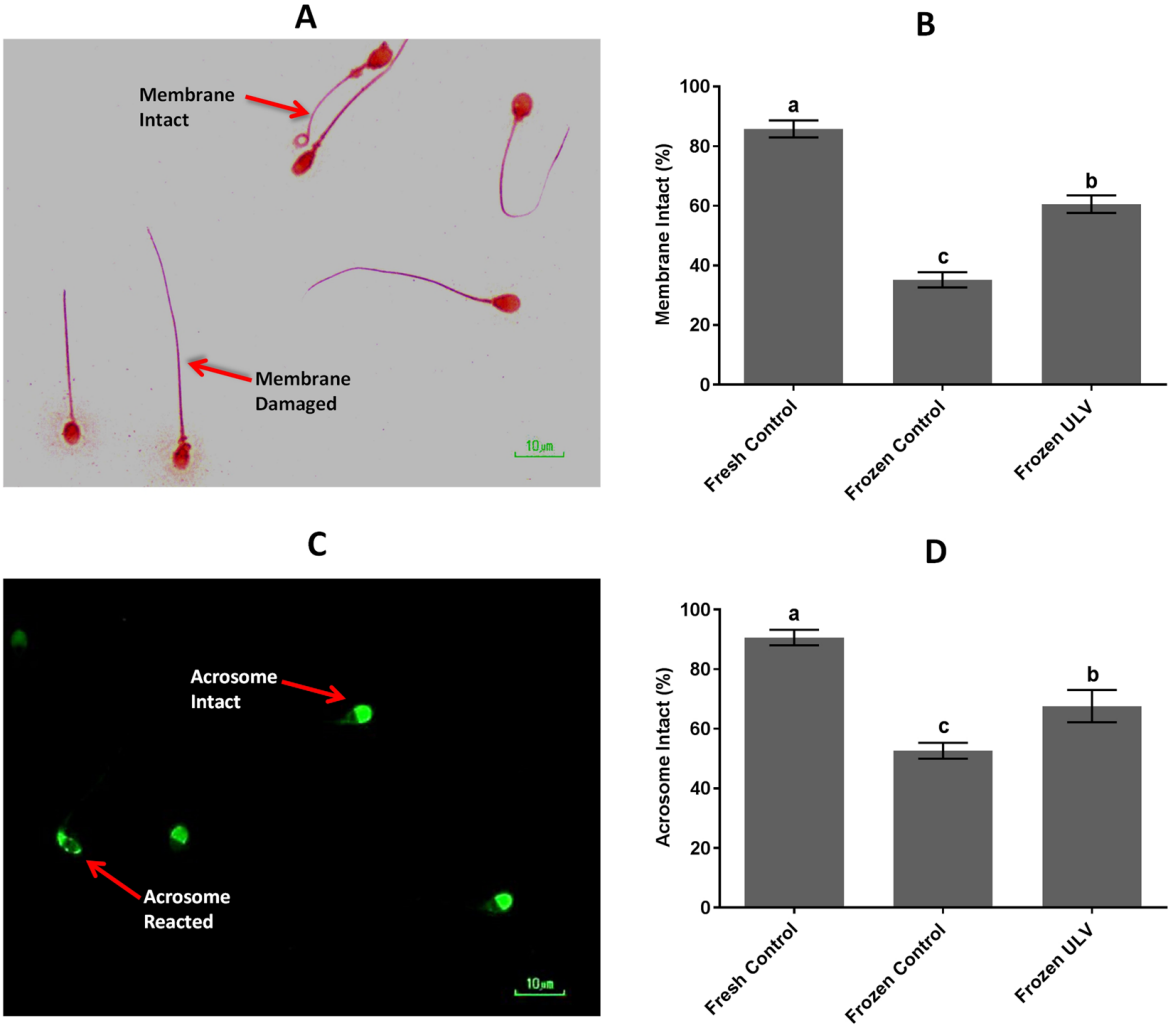
HOST and PSA. (**A**) The membrane intact and damages of sperm are apparent. (**B**) The rate of membrane intact was significantly altered in response to ULV exposure. (**C**) Sperm with intact and reacted acrosome were stained with FITC/PSA. (**D**) The percentage of sperm with intact acrosome was compared in three groups. The following letters a, b and c denote significant differences (p < 0.001). Values are represented as the mean ± SD; Tukey’s post hoc test; n = 25.

Figure 3 demonstrates the membrane integrity and acrosome integrity of sperm using the hypo-osmotic swelling test (HOST) and FITC/PSA methods, respectively. The rate of membrane intact was significantly (p < 0.001) higher in the fresh control (85.80 ± 2.84%) and frozen ULV (60.56 ± 2.93%) in comparison with the frozen control (35.16 ± 2.93%). The difference between the fresh control and frozen ULV was also statistically significant (p < 0.001).

Additionally, the acrosome intact was significantly (p < 0.001) higher in the fresh control (0.60 ± 2.61%) and frozen ULV (67.64 ± 2.41%) than that of the frozen control (52.64 ± 2.64%). This index was significantly altered between the fresh control and frozen ULV (p < 0.001).

**Figure 4:**
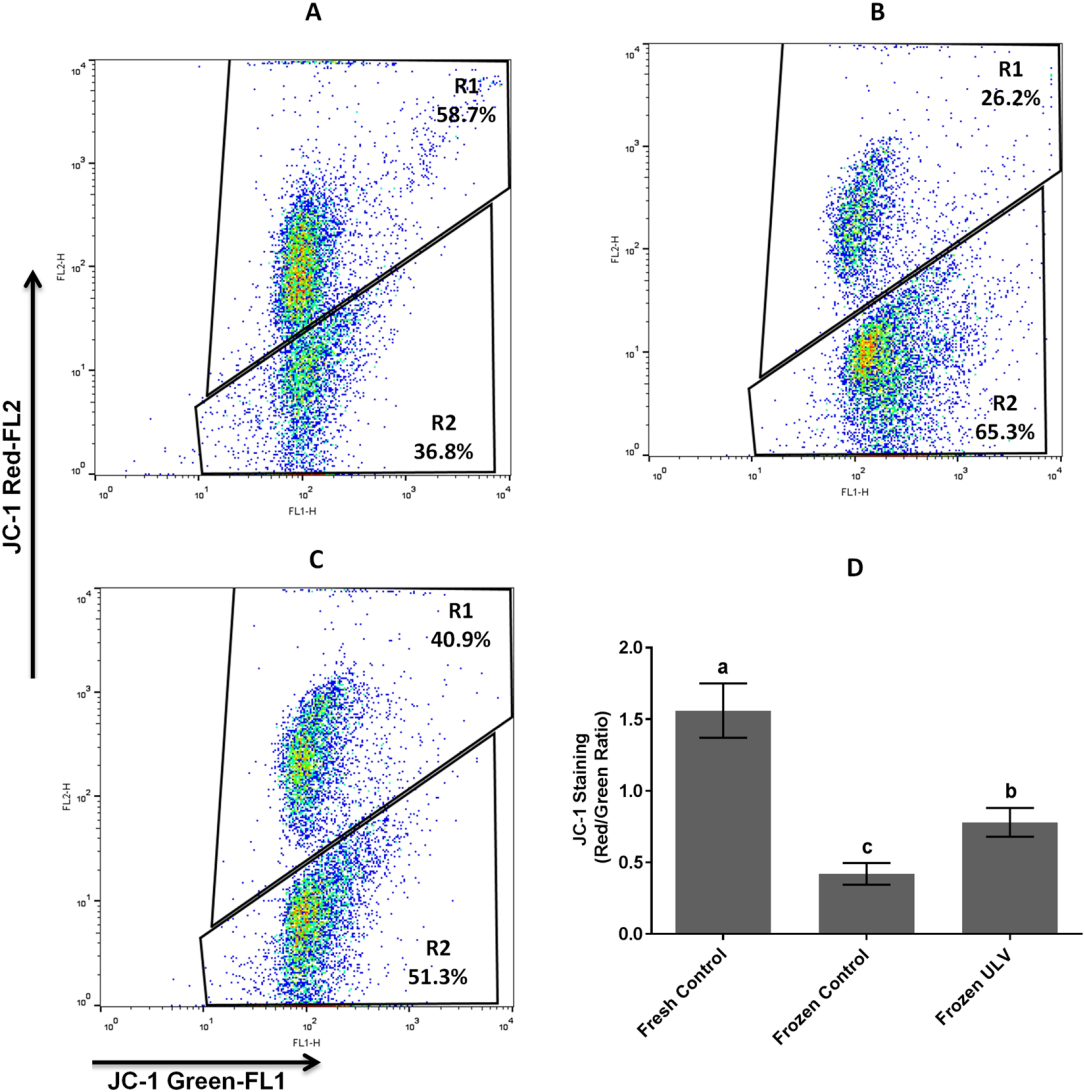
Mitochondrial membrane potential. The flow cytometry analysis of sperm in the fresh control (**A**), frozen control (**B**) and, frozen ULV (**C**) stained with JC-1. The graph shows the ratio of red to green fluorescent dye indicating the index of mitochondrial membrane potential in all experimental groups. (**D**) The top right quadrant represents the percentage of values demonstrating the proportion of sperm with high mitochondrial membrane potential while the bottom right quadrant indicates the proportion of sperm cells with low mitochondrial membrane potential. The following letters a, b and c denote significant differences (p < 0.001) among the groups. Values are expressed as the mean ± SD; Tukey’s post hoc test; n = 25.

The mitochondrial membrane potential was displayed in Fig. 4. The ratio of red to green fluorescence was significantly (p < 0.001) increased in the fresh and frozen ULV (1.56 ± 0.19 and 0.78 ± 0.78, respectively) in comparison with the frozen control (0.42 ± 0.08). This ratio was significantly higher in the fresh control than that of the frozen ULV (p < 0.001).

### Sperm DNA integrity

**Figure 5:**
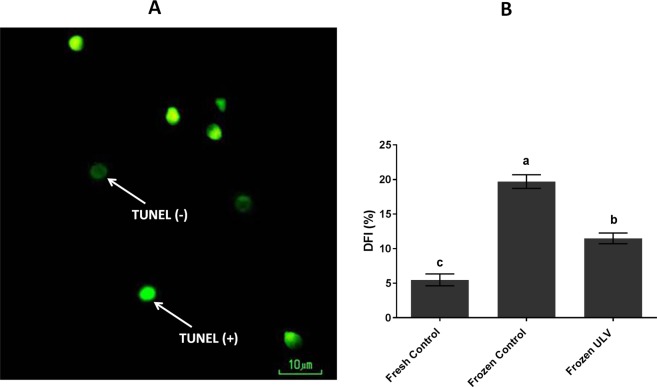
DNA fragmentation of sperm using the TUNEL assay in all experimental groups. (**A**) Sperm with intact DNA is visible as light green (TUNEL negative) while sperm with damaged DNA is indicated as bright green (TUNEL positive) under fluorescent microscopy at 100× magnification. (**B**) The graph shows the DNA fragmentation in all experimental groups. The following letters a, b and c imply significant differences (p < 0.001) Values are expressed as the mean ± SD; Tukey’s post hoc test; n = 25.

The labeled sperm demonstrated that the DNA fragmentation index was significantly reduced (p < 0.001) in the fresh control and frozen ULV (5.48 ± 0.85 and 11.48 ± 0.78%, respectively) when compared with the frozen control (19.70 ± 1.00%). Moreover, this value was significantly (P < 0.001) altered between the fresh control and frozen ULV (Fig. 5).

### Apoptosis assay

**Figure 6:**
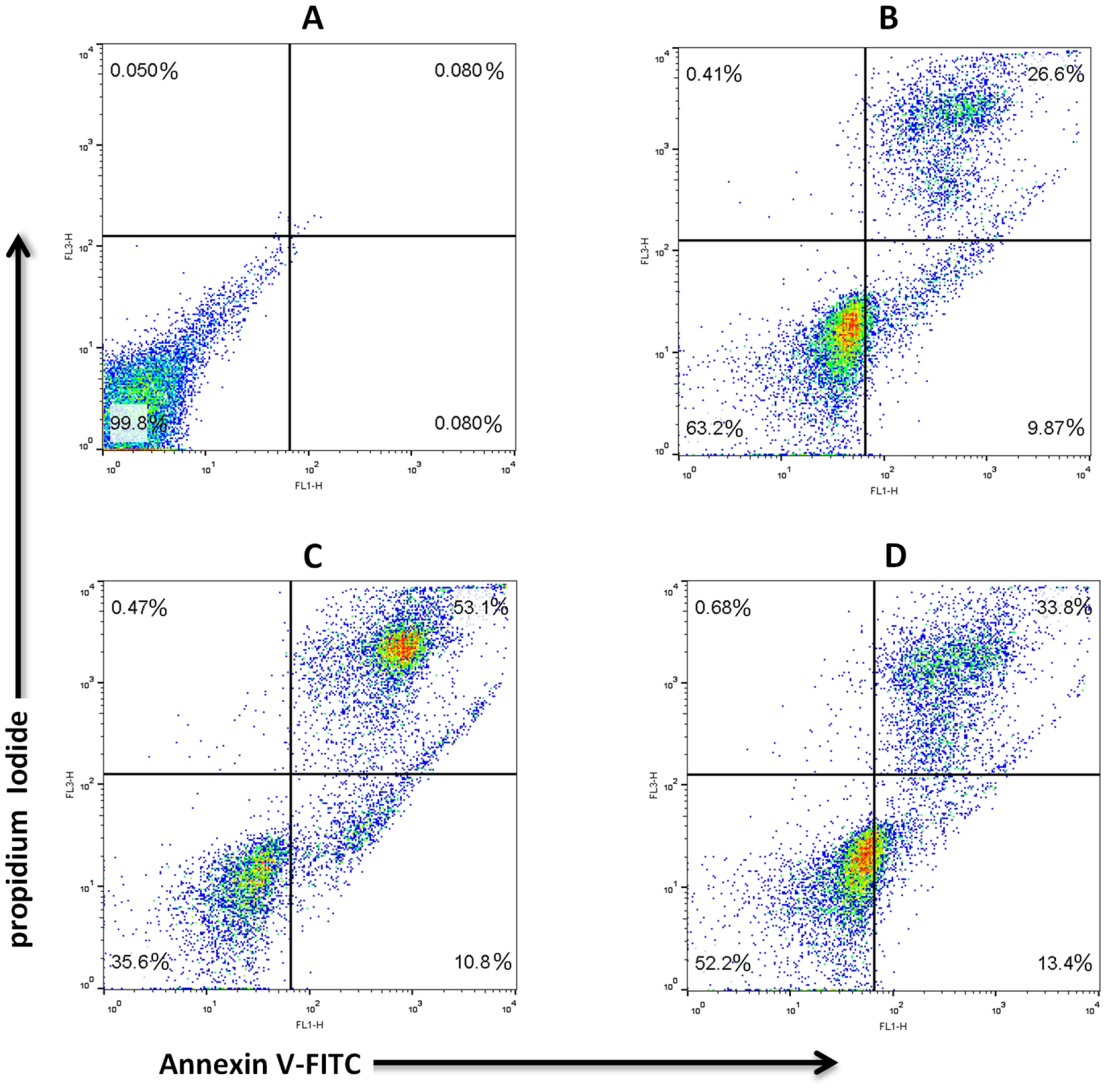
Apoptosis rate in human sperm. Unstained sperm is represented in the panel (**A**), represented as the fresh control (**B**), frozen control (**C**), and frozen ULV(**D**). The left top quadrant indicates necrotic cells, the left lower quadrant exhibits live cells, and the right lower and the right top quadrants represent the early apoptotic and late apoptotic cells, respectively.

As shown in Fig. 6 and Table 2, according to the pattern of cell staining with Annexin V (An) and propidium iodide (PI) sperm are categorized into four cell population. The number of live sperm (An^−^/PI^−^) was significantly lower (p < 0.001) in the frozen control in comparison with the fresh control and frozen ULV. The percentage of early apoptotic sperm (An^+^/PI^−^) was significantly higher in the frozen control than the fresh control and frozen ULV. The percentage of the late apoptotic sperm (An^+^/PI^+^) was significantly higher in the frozen control compared with the fresh control and frozen ULV. These three values were significantly altered between the fresh control and frozen ULV (p < 0.001). The percentage of necrotic sperm (An^−^/PI^+^) was significantly higher (p < 0.001) in the frozen control than that of the fresh control and frozen ULV, while there was no significant difference between the fresh control and frozen ULV (p > 0.001) for this value.

**Table 2:** Staining sperm with Annexin V and propidium iodide (PI). The following assigned letters a, b and c imply significant differences (P < 0.001) among the groups. Values are expressed as the mean ± SD; Tukey’s post hoc test; n = 25. An^−^/PI^−^, normal (viable) cells; An^+^/PI^−^, early apoptotic cells; An^+^/PI^+^, late apoptotic cells; An^−^/PI^+^, necrotic cells.

Sample	N	An^−^/PI^−^ (%)	An^+^/PI^−^ (%)	An^+^/PI^+^ (%)	An^−^/PI^+^ (%)
Fresh Control	25	69.73 ± 1.06^a^	9.62 ± 0.3^c^	20.10 ± 1.01^c^	0.54 ± 0.03^b^
Frozen Control	25	35.09 ± 0.43^c^	15.36 ± 0.46^a^	47.99 ± 0.73^a^	1.54 ± 0.44^a^
Frozen ULV	25	51.79 ± 0.3^b^	11.89 ± 0.47^b^	27.55 ± 0.36^b^	0.85 ± 0.03^ab^

### mRNA expression of tubulins and TPPP

**Figure 7:**
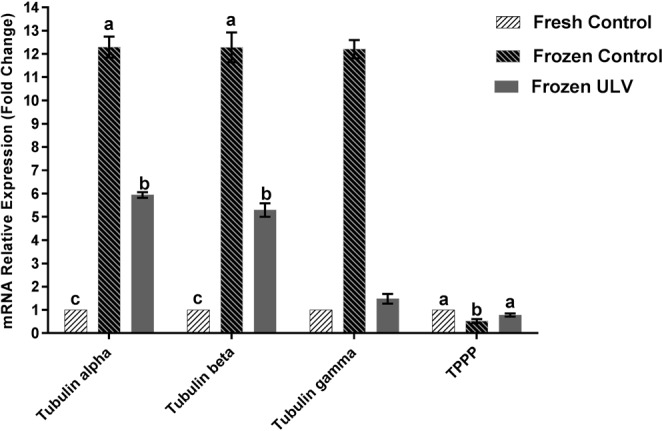
The bar plot indicates the mRNA expression of the various types of tubulins and TPPP genes in all experimental groups. The expression ratios of α-tubulin and β-tubulin were significantly increased in the frozen control compared with the fresh and frozen ULV groups. The mRNA expression of the TPPP was significantly reduced in the frozen control in comparison with the fresh control. The alteration in the mRNA expression of γ-tubulin was not significant among all groups of the study. Data were expressed as mean ± SD and the following assigned letters a, b and c denote significant differences (p < 0.001) among all groups; Tukey’s post hoc test; n = 25.

The results demonstrated that the gene expression levels of α- and β- tubulins were markedly higher in the frozen control group (p < 0.001) in comparison with the fresh control (12.29 ± 0.45 vs. 1.0 ± 0.00 and 12.28 ± 0.64 vs. 1 ± 0.00 folds, respectively) and frozen ULV (12.29 ± 0.45 vs. 5.94 ± 0.12 and 12.28 ± 0.64 vs. 5.29 ± 0.29 folds, respectively). The levels of the gene expression for α and β- tubulins were significantly higher in the frozen ULV when compared with the fresh control group (5.94 ± 0.12 vs. 1.00 ± 0.00 and 5.29 ± 0.29 vs. 1.00 ± 0.00 folds, respectively). Moreover, the mRNA expression of the TPPP gene was diminished by 1.92 ± 0.08 folds in the frozen control in comparison with the fresh control. The expression of TPPP was also decreased by 1.28 ± 0.065 folds in the frozen groups when compared with the frozen ULV (p < 0.001). The mRNA expression of TPPP was not significantly different between the frozen ULV and fresh control. Of note, the expression of γ- tubulins remained unchanged when compared among all studied groups (Fig. 7).

### Enzyme-linked immunosorbent assay (ELISA)

**Figure 8:**
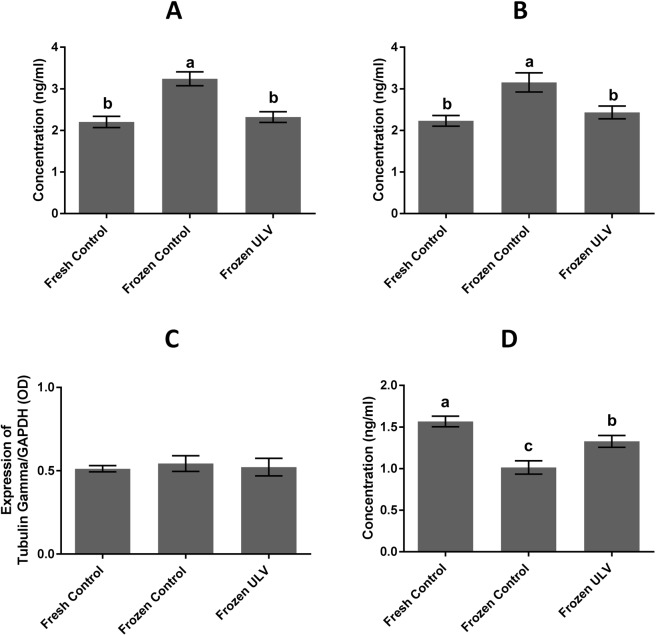
The concentrations of the sperm tubulin proteins evaluated by the ELISA method. The levels of α, β and γ-tubulins were assayed by the ELISA method in all groups. The concentrations of α and β tubulins were significantly increased in the frozen control compared with the fresh and frozen ULV groups (A and B panel, respectively). The level of γ-tubulin remained unchanged (**C**) while the level of TPPP was significantly diminished in the frozen control compared with the fresh and frozen ULV (**D**). Error bars represent the standard deviation (mean ± SD). The following assigned letters a, b and c denote significant differences (p < 0.001) among the groups; Tukey’s post hoc test; n = 25.

The concentrations of α and β tubulin at the protein level were assayed by the ELISA method. The results showed that α and β tubulin levels were significantly (p < 0.001) increased in the frozen control (3.24 ± 0.16 and 3.15 ± 0.23 ng/ml, respectively) in comparison with the fresh control (2.20 ± 0.13 and 2.23 ± 0.12 ng/ml, respectively) and frozen ULV (2.31 ± 0.12 and 2.43 ± 0.15 ng/ml, respectively), while the level of the above proteins were not significantly altered in the frozen control and frozen ULV. The level of the TPPP was significantly decreased in the frozen control (1.01 ± 0.08 ng/ml) as compared to the fresh control and frozen ULV (1.56 ± 0.06 and 1.32 ± 0.07 ng/ml). This expression of TPPP was also significantly (p < 0.001) higher in the fresh control than that of the frozen ULV. However, the level of γ-tubulin remained at the baseline level in all experimental groups (Fig. 8).

## Discussion

It has been implicated by numerous studies that magnetic, electromagnetic fields and ULV influence the physicochemical properties of the water molecules such as viscosity, density, pH, zeta potential, mean bubble size, etc.^11,13–15^. In the present study, we evaluated whether the freezing medium that contained ULV-exposed water could be used for the enhancement of human sperm cryopreservation.

We proposed that ULV is capable of modifying the physicochemical properties of water and it could interfere with the regular shape of ice crystals that might lead to fewer cryo-damages to sperm during the cryopreservation process. Recent reports indicated beneficial effects of magnetic field on the cryopreservation media, as well as the improvement in fertility potential of thawed sperm in boar and rooster^16,17^. In a study conducted by Lee *et al*., they showed that magnetized extender enhanced the quality of post-thaw sperm through a reduction in ROS and an increase in the antioxidant capacity of sperm cells^16^. Moreover, Askarianzadeh *et al*. suggested that magnetic field can lower the size of ice crystal after exposure of cryopreservation medium to the magnetic field^17^. However, to date, there is no report on the impacts of ULV on the freezing media used for human semen cryopreservation.

In the present study, double-distilled water was subjected to ULV at the different time periods, and then the physicochemical properties of the water molecules were determined to select the optimal ULV exposed water sample for being incorporated into the freezing media used for human sperm cryopreservation. The first step of the study was the measurement of the created bubble size in the water. Molecular basis of dynamic light scattering (DLS) method is laser radiation to the samples and the detection of the amount of light scattering emitted from the molecular structures in the solution. Since the DLS method requires an aquatic medium, solid substances must be first dissolved in water to be able to exhibit light scattering. Considering that solubility is not defined for the water, ULV was regarded as the sole variable in our experiment. As illustrated in Fig. 1A, different mean bubbles sizes (nm) were found in samples containing ULV-exposed water molecules. Previous studies suggested that acoustic wave could influence the cavitation bubble size in the water^18^. Two types of ultrasonic cavitation including inertial cavitation and non-inertial cavitation are found in the water molecules. The inertial bubbles collapse sharply, and their lifetime is less than one-tenth of a second. The non-inertial bubbles created at the frequency of 40 kHz possess much larger size compared to inertial bubbles. Furthermore, the lifetime of non-inertial bubbles is more than three minutes^18,19^. Consistent with previous reports, our findings showed that after exposure of the water molecules to ULV, the mean size bubble was significantly enlarged compared with control samples^18,20,21^.

The second assessment in our experiment was the determination of zeta potential. This parameter is an essential index of colloidal stability. In molecules and small particles, high values of zeta potential (negative and positive) are electrically stable, and solutions are resistance to aggregation. Zeta potential is used for monitoring of the behavior of colloidal solutions, and it was significantly increased in response to that treatment of the water molecules with ULV at various time periods (Fig. 1B). Therefore, the impact of ULV on the induction of colloidal structures present in the water was assessed. Notably, the negative values of zeta potential in the bubbles are due to the presence of hydroxyl groups in the bubble interface^22^.

The third parameter of the physicochemical properties of water was the measurement of the pH shown in Fig. 1B. The pH of samples was elevated in parallel with the increase in the time periods. Our findings confirmed that the pH of the water along with zeta potential is significantly increased. Yoshi Kubo *et al*. showed that zeta potential and high negative charge of samples measured at the air-bubble interface was due to the absorption of hydroxyl ions at this region resulting in high negative values of zeta potential in samples. Our data showed that in the low values of pH, the negative hydroxyl ions which are naturalized by the positive protons could decrease zeta potential that is in agreement with the findings of Yoshi Kubo *et al*. who reported that in the low pH (<4), the negative hydroxyl ions are neutralized by positive protons in the aqueous medium^23^.

Another parameter measured in our investigation is the electric conductivity. Our analyses demonstrated that the difference in electric conductivity was not statistically meaningful when the groups were compared. The increase in the electric conductivity could be related to the concentration of mineral salts and other electrolytes in the water. Since double distilled water was employed in this experiment, therefore, lack of change in electric conductivity in all types of samples would be seemingly logical. The values of the electric conductivity obtained from the water molecules (Fig. 1A) are in line with reports presented in previous studies^24^.

In the present experiment, the optimally treated water was obtained concerning the physicochemical properties of the samples exposed to ULV in a period of 18 minutes. Then, this condition was applied for being incorporated into the freezing media used for cryopreservation of human semen. We found that the quality of semen parameters is remarkably preserved upon the exposure of post-thaw sperm to ULV.

The improved quality of the post-thaw sperm that were cryopreserved in the freezing medium containing ULV-exposed water molecules could be attributed to the formation and growth of bubble cavitation^25^. It has been reported that ULV can initiate and induce the nucleation of ice in the water and supercooled aqueous solutions for the long-term period^26^ thought as the main reason for acoustic cavitation and subsequently the impairment of ice crystal morphology in terms of the shape and size distribution^27^. These changes in the formation of amorphous ice crystal result in the reduction of damages to sperm during cryopreservation. It should also be mentioned that in some reports direct exposure of sperm to ULV has detrimental effects on the sperm parameters such as count, percent motility, and normal morphology^28^. Thus, regarding the harmful impact of direct exposure of ULV on sperm cells, we exposed the freezing media to ULV before the dilution of sperm.

In the present study, the seminal concentration of ROS was assessed in frozen-thawed sperm. A significant reduction was detected in the presence of the freezing medium containing ULV-exposed water molecules. A large body of evidence indicated the deleterious effects of excessive ROS production on sperm functions^29,30^. Our findings were in line with the results obtained in the study of Sang *et al*. who claimed that magnetic field reduced ROS levels in boar semen^16^. Also, Hanaoka *et al*. demonstrated lower ROS production in response to the electrolysis of the water molecules^31^.

So, the higher percentage of motility and viability in frozen ULV may stem from the decreased ROS production. Also, another critical indicator of sperm quality which was significantly improved in response to ULV treatment was mitochondrial membrane potential. It seems that ROS can also directly affect mitochondrial membrane potential of sperm. It has been implicated in previous research that ROS can damage to the mitochondrial components, including proteins, lipids, and mtDNA^32^ that could subsequently change the signaling pathways, initiating the apoptotic cell death^33,34^.

Additionally, hydrogen peroxide, one of the primary pro-oxidant agents in oxidative damage, induces mitochondria fission within the cells^35,36^. Besides, superoxide anion is capable of causing mitochondrial fragmentation^37^. This event leads to a decrease in ATP production, and it subsequently lowers the energy supply for sperm motility^38,39^. Therefore, ROS stimulate the signaling pathways involved in mitochondrial fragmentation that is responsible for reducing the mitochondrial membrane potential and increasing the rate of apoptosis. The other detrimental impacts of ROS originate from its effects on DNA integrity. ROS produces 8-Oxo-2′-deoxyguanosine which is considered one of the main oxidized byproducts generated during DNA oxidation, leading to DNA single-strand breakage^40^. This parameter was significantly lower in the frozen ULV implying the inhibitory effects of ULV-exposed water (in freezing media) on ROS production during the cryopreservation process.

In the present study, the population of apoptotic sperm (An^+^/PI^−^ and An^+^/PI^+^) was reduced in the frozen ULV. This phenomenon was also corroborated by the earlier studies^41,42^ which suggested that the hindrance of mitochondrial fission could cause higher mitochondrial membrane potential which prevents the release of cytochrome c and decreases the rate of apoptosis^43,44^. Therefore, it seems that the reduction in ROS generation is accompanied by the improvement in mitochondria membrane potential and delayed apoptosis in the presence of the freezing medium containing ULV-exposed water.

Cryopreservation may lead to significant changes in the expression of a group of genes at the transcription and/or translation stage that could affect the quality of sperm^45,46^. The question is that which types of proteins and/or genes might be affected and when they are expressed or degraded within sperm^47,48^. In this experiment, we observed significant upregulation in the α and β-tubulin genes and proteins in the frozen control compared with other experimental groups. Therefore, these data demonstrated that alterations in the gene expression could occur during cryopreservation as this idea was validated by previous investigations^45^.

In this study, an increase in the expression of α and β-tubulin during cryopreservation could be extrapolated in several aspects. One of the reasons for the increase in the expression of α and β-tubulins might be ascribed to the higher concentrations of these types of tubulins in comparison with other tubulins, elucidating why they are more prone to be affected by environmental conditions such as cold stress. These results are in line with previous studies, indicating irregularities in the expression of α and β- tubulin are increased during cryopreservation of human sperm^30,49^. Since the concentration of α-tubulin is higher than other tubulins, it is more susceptible to damages caused by the freezing process. Another reason for an increase in the levels of α- and β-tubulins is microtubule depolymerization during cryopreservation^30^. In this case, α- and β-monomers of tubulins are exposed so that their epitopes are readily available to specific antibodies, and their expression would be clearly detectable in the ELISA method. Therefore, we propose the further investigations on these proteins and their protective roles in the functionality of sperm.

The decrease in TPPP was clearly observed in cryopreserved samples. Since TPPP is directly involved in microtubule polymerization^50,51^, microtubule bundling, stabilization of microtubule ultra-structures, and the integrity of the microtubule networks^52^, a reduction in the expression of TPPP is considered a typical event during cryopreservation. So, the reduction of TPPP could decrease the stability of microtubules, resulting in the impairment of sperm motility and fertility potential.

Our data demonstrated that γ-tubulin did not significantly change during cryopreservation in all experimental groups. Perhaps, one of the reasons for these observation is the lower concentration of γ-tubulin in comparison with α and β-tubulin that it is required for microtubule polymerization^30^. Previous reports stated that γ-tubules are resistant to cold stress, and drugs which cause the induction of depolymerization of microtubules^53^. Therefore, γ-tubulin is seemingly more conserved in comparison with other tubulin species^54^, and it is not significantly altered during cryopreservation in all experimental groups when analyzed at the gene and protein levels. To our knowledge, this is the first study that evaluated the impacts of cryopreservation on the regulation of tubulins and TPPP expression in the presence/absence of ULV.

It is concluded that ULV changes the physicochemical characteristics of the water. ULV induces the ice nucleation and causes lower damages to sperm during cryopreservation. We also showed that the application of a freezing medium containing ULV-exposed water could improve the quality indices of post-thaw human sperm. All in all, it seems further studies are warranted to illuminate the mechanism underlying the beneficial impacts of ULV on sperm parameters.

## Material and Methods

### Chemicals and ethics

All chemicals used in this experiment were purchased from Sigma (St. Louis, MO, USA) unless mentioned otherwise. All volunteers were adequately informed about the usage of their clinical and biological data for the experiments before giving their consent. The study was approved by the Research Ethical Committee of the Royan Institute, Tehran, Iran (http://ethics.research.ac.ir/IR.ACECR.ROYAN.REC.1397.033), and it was conducted according to the ethical guidelines of the Helsinki Declaration.

### Study design and ultrasonic exposur**e**

In this experiment, 300 W ultrasonic power generated by the ultrasonic water bath was used at the frequency of 28 kHz. The details were presented in Supplementary Information.

### Measurement of the mean bubble size, zeta potential, pH, and electric conductivity

The mean bubble size and zeta potential were determined by the DLS instrument (Brookhaven Instruments Corporation, USA). Furthermore, pH and electric conductivity of the water samples that were subjected to ULV was measured (control samples were not exposed to ULV). Further details of the experimental procedures were summarized in Supplementary Information.

### Human semen cryopreservation with a freezing medium containing ULV-exposed water molecules and the preparation of freezing media

Based on the physicochemical results of water samples, the optimum condition was selected for the preparation of the freezing medium. For the preparation of N-tris (hydroxymethyl) methyl-2-aminoethanesulfonic acid + tris (TEST) in the frozen ULV, the water was exposed to ULV for 18 minutes and the basic TEST was set at 325 mOsm, and then the pH was adjusted to 7.5 before the addition of the egg yolk. Next, 20% egg yolk was mixed to TEST buffer and centrifuged at 1,000 g for 20 minutes. The supernatant was then filtered by a 0.45 μm Acrodisc syringe filter (Merck Millipore Company, U.S). Finally, 10% glycerol and 2% DMSO were added to the TEST medium for the following experiments.

### Semen collection, cryopreservation, and thawing

Semen samples were collected from 25 individuals (with normal morphology >4%, motility >40%, and sperm concentration of >20 × 10^6^/ml) according to world health organization (WHO) criteria^55^, with 3–5 days of ejaculatory abstinence. After primary semen analysis, the specimens were equally divided into three sets of aliquots and assigned as the following experimental groups; i) fresh control; the aliquot was not cryopreserved, ii) frozen control; the aliquot was cryopreserved with a regular freezing medium without the application of ULV, and iii) frozen ULV; the aliquot was cryopreserved with a freezing medium containing ULV-exposed water. For cryopreservation, the samples were liquefied at 37 °C for 30 minutes, and rapid freezing was performed according to the method of Jeyendran *et al*.^56^ with slight modifications. The samples were then thawed after two weeks. Further details were summarized in Supplementary Information.

### Motion characteristics

For the determination of motion characteristics of thawed sperm by computer-assisted sperm analyzer (CASA, Version 5.1; Microptic, Barcelona, Spain), 5 μL of specimen was loaded onto a pre-warmed 20 μm chamber (Leja 4, Leja Products Luzernestraat B.V., Holland) and the parameters of motility (%), progressive motility (%), VCL (µm/sec), VSL (µm/sec), VAP (µm/sec), LIN (%), STR (%), ALH (µm), and BCF (Hz) were analyzed.

### Viability and morphology

The SYBR14/PI staining was applied for the evaluation of viability using the Live/Dead Sperm Viability Kit (catalog # L-7011). Briefly, a 50-fold dilution of the SYBR-14 stock solution was prepared in a buffer solution (10 mmol/L HEPES, 150 mmol/L NaCl, and 10% bovine serum albumin, pH 7.4). Then, 5 µl of diluted SYBR-14 and 5 µl of propidium iodide were added to 1 ml of diluted semen and then incubated for 5–10 min at 36 °C. The samples were observed under a fluorescent microscope at 100× magnification at an excitation wavelength of 488 nm. When the SYBR-14/PI staining was excited at 488 nm, the viable cells (stained with SYBR-14) were evident as bright green while the dead cells (stained by PI) were apparent as red cells.

For the assessment of the abnormal morphology, the specimens were stained with Papanicolaou stain. Further details were summarized in Supplementary Information.

### Measurement of ROS, and MDA

The extracellular ROS levels were measured by the chemiluminescence assay using luminol (5-amino-2, 3-dihydro-1, 4-phthalazinedione; Sigma, USA) as a probe. The intracellular ROS was determined according to WHO criteria^55^ by 2′,7′-dichlorofluorescin diacetate (DCFH-DA), and DHE. Lipid peroxidation was assessed based on the MDA level using the thiobarbituric acid (TBA) method with slight modifications^57^. Details of the experimental procedures were presented in Supplementary Information.

### Membrane integrity, acrosome integrity, and mitochondrial membrane potential

According to WHO criteria, the HOST was carried out for the evaluation of the membrane integrity^55^. The percentage of acrosome integrity was investigated using fluorescein conjugated lectin Pisum sativum agglutinin (FITC-PSA) staining method according to WHO criteria^55^. Moreover, for the assessment of the mitochondrial membrane potential, the specimens were stained with 5,5′,6,6′-tetrachloro-1,1′3,3′-tetrathylbenzimidazolyl-carbocyanine iodide (JC-1) stain. Further details concerning the measurement of these values are summarized in the Supplementary Information.

### DNA fragmentation index and apoptosis

The DNA integrity of sperm was detected by the terminal deoxynucleotidyl transferase dUTP nick end labeling (TUNEL) assay using the *In Situ* Cell Death Detection Kit (Roche Diagnostic GmbH, Mannheim, Germany) according to manufacturer’s instructions and then evaluated under fluorescent microscopy at 100× magnification, followed by the count of at least 200 sperm nuclei, in which the intactness of DNA was represented as the light green (TUNEL negative), and damaged DNA was depicted as the bright green color (TUNEL positive). The rate of the light green head and bright green head sperm were determined as well.

The apoptosis index was evaluated using the phosphatidyl-serine detection kit (IQ Products BV, Rozenberglaan 13a 9227 DL Groningen, the Netherlands). Further details are summarized in Supplementary Information.

### Density- gradient centrifugation

The removal of the seminal plasma was carried out using DGC for the elimination of other cells and contaminations including leucocytes, dead sperm, debris, etc.^58^ before the process of sperm cryopreservation. Briefly, 1 ml of 90% AllGrad [900 µl of 100% AllGrad (Life Global^®^ Media, USA) and 100 µl of HTF was gently placed in a test tube and then slowly poured on 1 ml of 45% AllGrad (450 µl of 100% AllGrad and 550 µl of HTF). Then 1 ml of semen sample was gradually put on the column AllGrad and centrifuged at 1500 rpm for 15 minutes. Then, 3 ml of HTF was added to the pellet and immediately after pipetting, the contents of the tube were centrifuged at 3000 rpm for 5 minutes. Afterward, 1 ml of PBS was added to the pellet, and pipetting was slowly performed. Next, the samples were resuspended and centrifuged at 3000 rpm for 5 minutes. The resultant pellet was employed for RNA extraction and protein expression assays.

## Real-time PCR

### RNA extraction and cDNA Synthesis

The cell suspension that underwent DGC was transferred into a 1.5 ml free RNase vial and 250 μl of TRIzol reagent (Cat # T9424, Sigma, USA) was added. Then, the resultant suspension was pipetted for 10 minutes on ice. Afterward, 50 μl of chloroform was added to the solution and vigorously agitated by vortex at 4 °C for 10 minutes. The mixture was left for 10 minutes on ice and then centrifuged at 12000 g at 4 °C for 15 minutes. Next, the upper phase was transferred into a new RNase-free microtube. Then, 100% isopropanol and 5 μl of glycogen were added and mixed with the supernatant and incubated at −20 °C overnight. The mixture was centrifuged at 12000 g at 4 °C for 15 minutes, and the pellet was washed using 75% ethanol. Then, the specimens were then centrifuged at 7500 g at 4 °C for 8 minutes. The resultant pellet was air-dried, and then 20 µl of RNase free water was added. The quantity of extracted RNA was determined using a NanoDrop instrument model One C (Thermo Fisher Scientific, USA) at a wavelength of 260 nm. For RT-PCR, 80 ng of total RNA was converted into cDNA using the cDNA Synthesis Kit (TAKARA, USA) based on manufacturer’s protocols and the obtained cDNA was stored at −20 °C for next use.

### Real-time PCR analysis

The design of the primers was carried out utilizing the Oligo7 software version 7.56 and by means of the RefSeq sequences of the GenBank database as listed in Table 3. The specificity of each primer pair was confirmed by the Primer-BLAST online software. Real-time PCR (on a Step One plus Real-time PCR system) was done for semi-quantification of the mRNA expression for genes mentioned earlier in a final volume of 10 µl, containing the SYBR Green Real-time PCR Master Mix (SYBR® Premix Ex Taq, Cat # RR820L, TAKARA, USA), 10 pmol of each primer, and 25 ng cDNA. The Real-time PCR protocol was implemented as follows: initiation denaturation at 95 °C for 600 seconds, denaturation at 95 °C for 15 seconds, annealing and extension at 60 °C for 60 seconds, followed by 40 cycles. Samples were normalized against the expression of β-actin as the internal control. The identification of fluorescent intensity in each sample was performed by the Step One™ Software version v2.3. The melting curve analysis showed only one peak for each reaction and confirmed by electrophoresis of PCR products.

**Table 3:** Primers used in Real-time PCR for the assessment of tubulins, TPPP, and internal control genes.

Locus	Primers	Amplicon size (bp)	Accession number
β-actin (F)	5′-AGACGCAGGATGGCATGGG-3′	161	NM_001101.3
β-actin (R)	5′-GAGACCTTCAACACCCCAGCC-3′	161	NM_001101.3
Tubulin α (TUBA1) (F)	5′-TGATGGAGCCCTGAATGTTG-3′	155	NM_032704.4
Tubulin α (TUBA1) (R)	5′-AGCAAGCATTGGTGATCTCTG-3′	155	NM_032704.4
Tubulin β (TUBB2) (F)	5′-CTGGATGTGGTGAGGAAGGAG-3′	163	NM_001310315.1
Tubulin β (TUBB2) (R)	5′-GCATGACGCTGAAGGTGT-3′	163	NM_001310315.1
Tubulin γ (TUBG2) (F)	5′-AGGACAACTTTGATGAGATGGAC-3′	147	NM_001320509.1
Tubulin γ (TUBG2) (R)	5′-CATCTAGAAGGAGAAGGAGTAGTGG-3′	147	NM_001320509.1
TPPP (F)	5′-CACTCTCCCTCCACACTTCC-3′	217	NM_007030.2
TPPP (R)	5′-GGTGACCACTATGTCCTCGT-3′	217	NM_007030.2

### Enzyme-linked immunosorbent assay

ELISA was used to quantify the protein levels of α, β and γ- tubulins and also TPPP in sperm in all experimental groups. The human α-tubulin (Cat # SEE714Hu), β-tubulin (Cat # SEB870Mi), γ-tubulin (Cat # ABIN1381574) and TPPP ELISA kit (Cat # SEA993Hu) were used to measure the total concentration of proteins mentioned above following the manufacturer’s instructions. The OD of samples was read at 450 nm using an ELISA reader model Expert 96 (Asys Company, UK). For the evaluation of γ-tubulin by the ELISA method, mouse anti-GAPDH antibody was used to detect the internal positive controls for the normalization of the OD values obtained from the target protein. The negative controls were HRP-conjugated anti-rabbit IgG and HRP-conjugated anti-mouse IgG antibodies that were applied alone in different wells without the application of the primary antibodies.

### Statistical analysis

The analysis of the obtained data was performed by the SPSS software version 16 (SPSS Inc. Chicago, ILL). For the evaluation of the physicochemical properties of the water, size (n = 150), zeta potential (n = 150), pH (n = 150), and electric conductivity (n = 150) were compared among groups at the different time periods. An 18 min time period was then selected as an optimum time period for the subsequent analyses.

The comparison of semen analysis, as well as other cellular and molecular properties, was performed only in the presence of the freezing medium containing ULV-exposed water for 18 min among the fresh control, frozen control, and frozen ULV groups.

All comparisons in this experiment were made by one-way ANOVA, followed by Tukey’s post hoc test. The level of significance was accepted if the p-values were less than 0.001. The Real-time PCR data were expressed as the fold change deduced by the ∆∆CT method.

## Supplementary information

The Application of Ultrasonic Vibration in Human Sperm Cryopreservation as a Novel Method for the Modification of Physicochemical Characteristics of Freezing Media
