# Supplementary material for: The Application of Ultrasonic Vibration in Human Sperm Cryopreservation as a Novel Method for the Modification of Physicochemical Characteristics of Freezing Media
Source: Sci Rep. 2019 Jul 11;9:10066. doi: 10.1038/s41598-019-46424-0 (PMC6624318; doi:10.1038/s41598-019-46424-0)
Supplement: Supplementary file 1 — The Application of Ultrasonic Vibration in Human Sperm Cryopreservation as a Novel Method for the Modification of Physicochemical Characteristics of Freezing Media [file 41598_2019_46424_MOESM1_ESM.pdf]

The Application of Ultrasonic Vibration in Human Sperm Cryopreservation as a Novel Method for the Modification of Physicochemical Characteristics of Freezing Media

Gholami D[1,2], Riazi GH[1], Fathi R[2], Ghaffari S.M[1], Shahverdi AH[2]*, Sharafi M[3]*, Alaei L[1]

[1]Institute of Biochemistry and Biophysics (IBB), University of Tehran, Tehran, Iran

[2]Department of Embryology at Reproduction Biomedicine Research Center, Royan Institute for Reproductive Biomedicine, ACER, Tehran, Iran

[3]Department of Poultry Sciences, Faculty of Agriculture, Tarbiat Modares University, Tehran, Iran

*** Corresponding authors:**

Abdolhossein Shahverdi

Royan Institute for Reproductive Biomedicine. No.2, Hafez St., Banihashem St., Resalat Ave., Tehran, Iran; P.O.Box: 16635-148; Tel: +98-21-22339940; Fax: +98-21-23562677, Email: shahverdi@royaninstitute.org

Mohsen Sharafi

Department of Animal science, Tarbiat Modares University (TMU), Tehran, Iran. P.O. Box: 14115-336, Phone No: +98 (021) 48292348. Email: m.sharafi@modares.ac.ir

Experimental design and ultrasonic exposure. The temperature of the water was set to 25 °C by circulating water through a closed-loop cooper piping system in the bath. Glass cells containing double distilled water (prepared by Double Distiller GFL model 2108, Burgwedel, Germany) was placed in the center of the bath, and the water bah was then covered and turned on. The temperature gradient above the surface of the waterline and the water available at the depth of the bath must be minimizing and does not change. In addition, the distance between the cover and the waterline in the bath must be about 60 millimeters, and the temperature of the area above the water should be the same with the water temperature. Five levels of time periods (0, 6, 10, 14, 18, and 22 minutes) were applied in ultrasonic water. The temperature was monitored using a chromel-alumel thermocouple, during the electromagnetic treatment and $\Delta\theta$ was < 0.01°C.

Mean bubble size, Zeta potential, pH and electric conductivity measurements. The mean bubble size was achieved based on the Stokes-Einstein equation [13]. For mean bubble size, 600 μl of sample water was loaded into cuvette chambers and illuminated using a 633 nm line of a HeNe laser and the signal was detected in a backscattering geometry and subsequently, the mean bubble size was determined by the DLS instrument and BIC Particle Sizing Software (Brookhaven Instruments Corporation, USA).

For the assessment of zeta potential, 1600 μl of water sample was loaded into cuvette chambers and zeta potential was measured based on the particle electrophoretic mobility by DLS and BIC PLAS Zeta Potential Analyzer Software (Brookhaven Instruments Corporation, USA).

pH of the water samples subjected and unsubjected with ultrasonic vibration was determined by pH meter (Pye Model 292, Pye Unicam). In addition, the electric conductivity of the water samples subjected and unsubjected with ultrasonic vibration was determined by Conductometer (PTI-8 Digital Conductivity Meter, Scientific Industries International Inc. UK).

Semen collection, cryopreservation, and thawing. For cryopreservation, the samples liquefied at 37°C for 30 minutes and rapid freezing was performed using Jeyendran et al. method [14] with slight modifications. Briefly, semen samples were slowly mixed with an equal volume of each medium. The mixture was then loaded into labeled cryovials and placed in liquid nitrogen vapor for 15 minutes and subsequently immersed in liquid nitrogen until the next analysis. According to the method of Jeyendran et al., frozen-thawed samples were diluted four-fold with Tyrode's salt solution. The diluted specimens were centrifuged at 800 g for 4 minutes at the room temperature. The sperm pellets were then slowly re-suspended in 0.5 mL of Tyrode's salt solution. The post-thaw sperm suspension was incubated at 37˚C in 5% CO2 for 20 min followed by sperm analysis.

Morphology. For assessment of the abnormal morphology, 20 μl of each sample was placed on a slide and air dried. The smears were then stained by Papanicolaou staining. Two hundred sperms were counted for each sample and percentages of abnormalities were determined by light microscopy at 100× magnification.

ROS and MDA measurements. For the evaluating ROS in semen, 10 μl luminol working solution (5 mM luminol prepared in DMSO) was added to 400 μl liquefied specimen (20×10^6 sperm/ml) and mixed gently. Positive controls were prepared by 395 μl PBS, 5 μl 30% H_2O_2 and 10 ul luminol working solution. Negative controls including 400 μl PBS and 10 μl luminol working solution. Chemiluminescence was assessed for 15 min using a luminometer and ROS was reported as RLU/Sec/10^6 sperm.

The intracellular ROS was determined by 25 μM DCFH-DA and 1.25 μM DHE which added to 1-3×10^6 sperm/ml fractions and fraction with DCFH-DA incubated at 25°C for 20 min and fraction with DHE incubated at 25 °C for 40 min in the dark room. Each sample was analyzed using a flow cytometer with a 488 nm argon laser (Becton Dickinson FACScan, San Jose, CA, USA). Green fluorescence of DCFH-DA (500-530 nm) and red fluorescence of DHE (590-700 nm) were evaluated with excitation wavelength at 488 nm and emission wavelength at 525-625 nm in the FL-2 channel. PI was used as a counterstain dye for DCFH for the distinction of dead sperm. Data for intracellular ROS were expressed as the percentage of fluorescent spermatozoa.

The MDA concentration was assessed in the seminal plasma. The specimens were centrifuged at 1500 g for 5 minutes and 1:2 ratio of TBA was added to the supernatant and incubated immediately at 95°C for 30 minutes and then allowed to cool on ice for 5 minutes. Afterward, the specimens were centrifuged at

1500 g for 5 minutes and the supernatant absorbance determined by spectrophotometer at the wavelength of 534 nm.

Membrane integrity. For measurement of membrane integrity, 1.351 gr of D-fructose and 0.735 gr of sodium citrate dihydrate were added to 100 ml of purified water to obtain a hypo-osmotic swelling solution. The semen (1:2 ratios) was mixed with the hypo-osmotic solution at 37°C for 30 minutes and the different HOST sperm-tail patterns were then counted by the light microscope at 100× magnification. A number of 300 sperm was randomly assessed to determine the percentage of swollen and non-swollen tails observed under a phase-contrast microscope at 400× magnification (CKX41, Olympus, Tokyo, Japan).

Acrosome integrity. A volume of 10 µl of the specimens was smeared on the microscope slide, dried and fixed in ethanol at 20°C for 30 min. The smear was stained with FITC-PSA and incubated at 4°C for 60 min. The numbers of 200 sperm was evaluated in each replicate using fluorescence microscopy at 100× magnification at 450-490 nm excitation.

Mitochondrial membrane potential. A 1mL of specimen adjusted to 3×10^6 cell/ml was stained with 1.0 µl of JC-1 stock solution (1.53 mM) for 15 minutes at 37°C and centrifuged for 5 min at 800 g. The resulting pellet was diluted 1:5 in PBS and measured for orange and green staining by flow cytometry. A total of 10,000 gated events based on the FS and SS were analyzed per sample using the flow cytometer for assessment of each specimen. A 488 nm filter was used to excitation of JC-1 and emission filters of 530 and 575 nm were used to quantify the population of spermatozoa with green and orange fluorescence, respectively. As shown in figure 3 Sperm with JC-1 staining was detected by FL1 with green color and FL2 with orange color canals.

Apoptosis. Specimens were washed in 1 ml calcium buffer 1X and centrifuged at 800 g for 5 minutes. The pellet was re-suspended in 1 ml calcium buffer 1X and the cell concentration adjusted to 1×10^6 sperm/ml. The 10 µl Annexin V- FITC was added and incubated for 20 minutes at 4°C and the cells were washed again with calcium buffer 1X. The 10 µl PI was then added to the specimens and incubated for at least 10 min at 4°C. The stained specimens were immediately analyzed using flow cytometry by measuring the fluorescence emission at 530 nm by FL1 canal and 575 nm by FL3 canal. Finally, we considered the normal sperm as (An^-/PI^-), early apoptotic sperm as (An^+/PI^-), apoptotic sperm as (An^+/PI^+) and necrotic sperm as (An^-/PI^+).
